# Examining the Effectiveness of Group Positive Parenting Training on Increasing Hope and Life Satisfaction in Mothers of Children with Autism 

**Published:** 2018-04

**Authors:** Seyed Omid Sotoudeh Navroodi, Mojgan Nicknam, Ameneh Ahmadi, Fatemeh Pooragha Roodbarde, Saeed Azami

**Affiliations:** 1 Roudehen Branch, Islamic Azad University, Roudehen, Iran.; 2 Department of Psychology, Roudehen Branch, Islamic Azad University, Roudehen, Iran.; 3 Psychiatry and Psychology Research Center, Tehran University of Medical Sciences, Tehran, Iran.; 4 Ehia Psychological Clinic, Rasht, Iran.; 5 Hoshiar Mental Health Institute, Tehran, Iran.

**Keywords:** *Autism Spectrum Disorder*, *Hope*, *Life Satisfaction*, *Positive Parenting*

## Abstract

**Objective:** Parents of children with autism spectrum disorders are exposed to mental distress because of having a disabled child more than parents with children with other psychological disorders, and their children's disorder has a negative effect on their hope and life satisfaction. The present study aimed to examining the effectiveness of group positive parenting training on increasing hope and life satisfaction in mothers of children with autism.

**Method** **:** This was a quasi-experimental study with pretest, posttest, and control and experimental groups. Mothers with autistic children (6-15 years) in Rasht consisted the statistical population of the study. All the children had a medical record and autism diagnosis based on DSM-IV-TR by a psychiatrist. Hope Questionnaires by Snyder and Life Satisfaction Questionnaire by Diener were implemented. Participants of the experimental group received positive parenting training for 8 sessions, and participants of the control group were put in the state of waiting. Descriptive statistics (mean, standard deviation, frequency, and percentage) and inferential statistics (univariate and multivariate covariance analysis) were used for data analysis.

**Results:** In this study, 27 mothers of children with autism were examined. The mean and standard deviation of the age of mothers in the experimental group was 36.14± 2.47 years and it was 37± 3.62 years for mothers in the control group. The results of univariate covariance analysis revealed a significant difference between the scores of pretest and posttest of the experimental and control groups in life satisfaction (Sum of square = 16.558, F = 13.534, DF = 1, P = 0.002, 〖=ƞ〗^2 = 0.361).

**Conclusion: **The results of this study showed that using group positive parenting training can have a positive effect on dimensions of hope and life satisfaction in mothers of children with autism spectrum disorder.

Autism spectrum disorder (ASD) is a developmental disability characterized by defects in social interaction, non-verbal communication skills, and repetitive behaviors (1). The prevalence of ASD has risen in the last years and the latest estimate by Center for Disease Control indicates that 1 in 68 children have been diagnosed with ASD (2).

 The average global prevalence of autism is 0.62% to 0.70% (3); however, the most recent large-scale surveys have estimated its prevalence to be 1% to 2% (4). A similar rate prevalence has been reported for adults alone (5). The number of reported cases of autism that have intellectual disability are about 45%, and 32% of the cases have regression (i.e., loss of already acquired skills; mean age of onset: 1.78 years) (6). 

Early studies showed that autism is seen in both genders, but it is 4 to 5 times more common in males than in females although the difference was prominent in individuals with intellectual disability (7). However, large-scale population-based studies (8) have also shown that autism affects males 2 to 3 times more than females. The fifth edition of the Diagnostic and Statistical Manual of Mental Disorders (DSM-5) has adopted the term “autism spectrum disorder” without a definition of subtypes and reorganized the triad into a dyad: social and communication deficits and restricted and repetitive behavior, interests, or activities. The delay or deviance in early language development was removed from the criteria, and it is now classified as a co-occurring condition even though the large variation in language is a characteristic of autism (9). Genetic predisposition, other biological factors and environmental influences, and their interactions are involved in the etiology of this disorder (10). Studies have shown that parents of children with autism spectrum disorder are more exposed to mental distress because of having disabled children than parents of children with other psychological disorders (11). These pressures can arise from incompatibility problem, non- sociable and self-harm behaviors, stereotyped movements, or mental pressure caused by the difficulty in establishing social contact and problems related to the presence in public places with special physical conditions of the child or high costs of education and health services (12). Pervasive developmental disorder is one of the disorders that causes many challenges for families (13). Despite many problems that parents experience for the birth of their child, the hope to have a healthy and natural child usually causes a feeling of confidence in parents and accepting the child (14). Mother is the first person who communicates directly with the child. Dealing with a child with mental retardation and disorder, the need to constant care, the importance of providing special facilities for growth, parents' experience of stress caused by ritualistic behavior, yelling language difficulties, and lack of self-care skills in this group of children provide grounds for undermining the normal functioning of the mother. One of the most important predisposing sources of this group of psychological problems is losing hope to life (15). Hope is considered as one of the coping resources of humans in adaptation to difficulties and even incurable diseases. Also, hope can be described as multidimensional dynamic powerful healing factor that can have an important role in adjustment to the trouble (16). For most families, this diagnosis is shocking and unexpected so that some parents reject the diagnosis and deny the existence of the disorder in their child. Autism spectrum disorder can predispose the parents to mental distress due to the following factors: (1) accompanying various language, communication, behavioral and social abnormalities; (2) difficulties in diagnosis; (3) incidence of the symptoms after the normal growth of the child; (4) lack of definite and effective treatments: and (5) unfavorable prognosis (17). Having meaning in life with interaction with hope can increase happiness, life satisfaction, and positive emotion and decrease depression (18, 19). Life satisfaction can be attributed to cognitive part of well-being and feelings about quality of life (20). Parents of children with ASD experience less psychological well-being than parents of children without ASD and other types of disabilities (21). Malta by et al. (22) found that individuals who have higher life satisfaction, use more effective and appropriate coping mechanisms, experience deeper positive emotions, and have higher general health. Lack of life satisfaction is correlated with weaker health condition, symptoms of depression, personality problems, inappropriate health behaviors, and poor social condition. There are many training programs for parents, each of which is different from the others in philosophy, methods, and effectiveness. In this regard, positive parenting program is a group behavioral intervention that is proposed by Sanders et al. (23,24). This program has 5 intervention levels because of existence of various levels in dysfunctions and various parent-child needs, and it is appropriate for parents of 12- to 13- year-old children that are suffering or are exposed to the risk of behavioral and emotional disorders. The present study aimed at preventing the incidence of behavioral, developmental, and emotional disorders in children by increasing the level of knowledge, skills, and self-confidence in parents (25,26). Parents-focused programs are developed with the aim of instructing effective methods to parents, so that they can instruct their children and reduce their challenging behaviors that are derived from their expansive needs; moreover, such programs can help parents evaluate their children's interfering behaviors appropriately. Also, parents' training programs increase parents' competence. A study by Tellgen and Sanders (27) showed that positive parenting program has a positive effect on parents' life style, self-efficacy, satisfaction, compatibility, relationships, and taking care of the child. Furthermore, a study by Hahlweg et al. (28) revealed that positive parenting program can reduce parenting stress and children's behavioral problems. Other studies have also shown that positive parenting can be used as a therapeutic method for clinical population and can also prevent intervention levels and cause significant decrease in children's behavioral problems permanently. Also, it results into consequences of parenting, such as decreasing disturbed parenting style, increasing parenting efficiency, and decreasing their stress (29). The result of a study indicated that positive parenting program can significantly reduce child maladaptive behaviors and improve parental and family functioning; and it may also be a feasible way to improve child, parent, and family functioning during the first year of ASD diagnosis (30). Evidence shows that mothers of children with autism spectrum disorder experience despair and decrease in life satisfaction. Thus, hope and life satisfaction can increase in mothers by providing therapeutic methods that can help them manage and control behavioral disorders. The present study aimed at examining the effectiveness of group positive parenting training on increasing hope and life satisfaction in mothers of children with autism spectrum disorder.

## Materials and Methods

This was a quasi-experimental study with pre-test, post-test, and control and experimental groups. Mothers of autistic children and adolescents (6-15 years old) in Rasht who referred to a psychological, speech therapy and occupational therapy clinic for treating their children in 2014, were enrolled in the study. All these children had a medical record and autism diagnosis based on DSM-IV-TR by a psychiatrist. A total of 27 individuals (experimental group (n = 14), control group n = 13)) were selected by convenience sampling method according to inclusion and exclusion criteria and randomly assigned into experimental and control groups. Inclusion criteria were as follow: a minimum of high school diploma degree, psychiatrist’s diagnosis of autism in their children, having an autistic child aged 6 to 15 years, and commitment to participate in therapy sessions. Exclusion criteria were as follow: use of other psychological treatments and having a child with other mental disorders in their family. Participants of the experimental group received positive parenting training for 8 sessions and participants of the control group were put in the state of waiting. 

**Snyder Hope Questionnaire: **This is a 12-item scale that has been designed by Snyder (31) for individuals older than 15 years old and has 2 subscales including crossing and motivation. To respond to each question, a continuum from 1 (totally wrong) to 4 (totally correct) is considered. In a study by Golzari that was conducted on 660 female students in Tehran, the reliability of this scale was examined using internal consistency method, and Cronbach’s alpha of 0.89 was obtained.

**Life Satisfaction Questionnaire:** Life Satisfaction scale was used in the present study. This scale was made by Diener et al. for all age ranges and was revised by Povet and Diener. It is rated on a 5-Point-Likert scale and each item can range from totally disagree (1) to totally agree (7), so that the possible range of the score varies from 5 (low satisfaction) to 35 (high satisfaction). Diener et al. in their study reported Cronbach’s alpha of 0.87 for the reliability of the scale and test-retest coefficient of 0.82 after 2 months. The reliability of Life Satisfaction Scale was examined by Bayani et al. on a population of 109 university students. In this study, the reliability of the scale was obtained to be 0.83 using Cronbach’s alpha and 0.69 using test-retest method. 

**The Method of Implementation and Analysis: **To gather and invite mothers of children with autism spectrum disorder, an announcement of forming group positive parenting training sessions specialized for mothers of children with autism spectrum disorder was prepared and was presented in all centers and counselling clinics, governmental and public centers, and occupational therapy and speech therapy centers in Rasht. After registering mothers and obtaining their phone number, 27 individuals were selected and randomly assigned into experimental and control groups (experimental group (n = 14) and control group (n = 13)). Positive parenting intervention sessions were presented in 8 two-hour sessions once a week for 2 months. Participants of the 2 groups were examined 2 times (pre-test and post-test) using Hope Questionnaire and Life Satisfaction Questionnaire. To analyze data, descriptive statistics (mean, standard deviation, frequency, and percentage) and inferential statistics (univariate and multivariate covariance analysis) were used.

***Intervention Sessions***

**First Session:** The followings were done in the first session: getting to know the members of the group; providing short explanation to the parents about their children's problems; emphasizing the rules; explaining autism spectrum disorder; and explaining positive parenting and the aim of using it; providing some reasons for behavioral problems in children; the way of recording children's behaviors; and giving tables for recording.

**The Second Session:** The followings were done in the second session: checking the assignments of the first session; providing feedback; reinforcing parent-child behavior; giving instructions; and presenting methods of training the skills and new behaviors.

**The Third Session: **The followings were done in the third session: checking the assignments and giving feedback; explaining the kinds of therein forcers; the way of giving verbal compliments; providing the main points of using rein forcers; and explaining the characteristics of the main principals governing the behavior of the children by parents.

**The Fourth Session:** The followings were done in the fourth session: checking the assignments and giving feedback; decreasing mild unfavorable behaviors; and explaining rational consequences and common problems in using the method.

**The Fifth Session:** The followings were done in the fifth session: checking the assignments and giving feedback; providing some guidelines for the survival of the family; diagnosing troublesome situations; and explaining the steps of the planned activities. 

**The Sixth Session:** The followings were done in the sixth session: checking the assignments and giving feedback and presenting the obstacles of keeping the changes.

**The Seventh Session:** The followings were done in the seventh session: encouragement; explaining the relationship of pleasant behavior and the kind of encouragement; presenting the effects of encouragement on behavior; the kinds of encouragement; and principles of encouragement.

**The Eighth Session: **The followings were done in the eighth session: Punishment; the aims of punishment, the methods of punishment; the negative effects of punishment; and principals of the punishment.

## Results

In this study, 27 mothers of children with autism (experimental group (n = 14) and control group (n = 13)) were examined. The mean and standard deviation of the age of mothers in the experimental group was 36.14± 2.47 years and was 37± 3.62 years for the control group. Also, the examination of the duration of marriage in case of year showed that the mean and standard deviation of the duration of marriage in the experimental group was 11.35±3.47 and it was 13.23±3.96 for the control group. The education level and occupational situation of the mothers were the other components that were examined. Analyzing the data showed that 42.9% of mothers in the experimental group had B.A. degree, 14.3% diploma, and 28.6% post-diploma degree and the rest had the degree higher than B.A. Also, 78.6% of the mothers in the experimental group were housewives. In the control group, 61.5% of the mothers had post-diploma degree, 23.1% B.A. degree, and the rest had a degree higher than B.A. Also, 76.9% of mothers were housewives. Presents the mean and standard deviation of hope dimensions and life satisfaction based on group and time of assessment (Table 1).

To examine the effectiveness of intervention on the total score of mothers' hope, univariate covariance analysis was used. Thus, first, the assumption of homogeneity of the variance of the error was examined using Leven test. The results of this test showed that there is no limitation in using this test (F_ (1, 25)_ = 3.151, P= 0.08). The results of univariate covariance analysis revealed a significant difference between the scores of pretest and posttest of the experimental and control groups in total score of hope (Sum of square= 40.544, F= 40.501, DF= 1, P= 0.0001, ƞ^2^= 628). To clarify the effectiveness of intervention on the total score of hope, multivariate covariance analysis was used to examine this intervention on dimensions of Hope Questionnaire (motivation and crossing). Thus, first, the assumption of homogeneity of matrix of variance and covariance were examined using M. Box test, and the results showed that this assumption is established (F_ (4_,_ 140813.213)_ = 0.732, P= 0.533, M. Box=2.406). Then, Leven test was used to examine the assumption of homogeneity of the variance of the error, and the results of the analysis showed that this assumption is established (Table 2). Thus, to examine the effectiveness of the intervention on dimensions of Hope Questionnaire, multivariate tests were used, and the results showed that intervention was effective on dimensions of Hope Questionnaire (motivation and crossing) (Lambdai Wilks=0.359, F=19.662, P=0.0001, ƞ^2^=641). Table 3 demonstrates the results of multivariate covariance analysis that examined the effectiveness of intervention on dimensions of Hope Questionnaire.

The results of multivariate covariance analysis showed that group positive parenting training increased dimensions of hope, such as motivation, in mothers of the experimental group. To examine the effectiveness of intervention on the level of life satisfaction, univariate covariance analysis was used and the assumption of homogeneity of the variance of the error was examined using Leven test. The results of this test showed that there is no limitation in using this test (F_ (1, 25)_ = 2.222, P= 0.14). The results of univariate covariance analysis revealed a significant difference between the scores of pretest and posttest of the experimental and control groups in life satisfaction (Sum of square = 16.558, F= 13.534, DF= 1, P = 0.002, ƞ^2^= 0.361).

**Table 1:** Mean and Standard Deviation of Dimensions of Hope and Life Satisfaction Based on Time and Group

**Component**	**Group**	**Time**	**Mean**	**Standard Deviation**
Motivation	Experimental	Pre-test	64/13	1/15
		Post-test	14/50	0/94
	Control	Pre-test	13/84	1/21
		Post-test	13/46	1/05
Crossing	Experimental	Pre-test	12/28	1/38
		Post-test	13/42	1/15
	Control	Pre-test	12/46	0/87
		Post-test	12/23	0/92
Total score of hope	Experimental	Pre-test	25/92	1/97
		Post-test	27/92	1/63
	Control	Pre-test	26/30	1/37
		Post-test	25/69	1/18
Life satisfaction	Experimental	Pre-test	20/92	1/49
		Post-test	22/57	1/65
	Control	Pre-test	20/65	2/01
		Post-test	21	1/77

**Table 2:** The Results of Leven Test to Examine the Assumption of Homogeneity of the Variance of the Error in Dimensions of Hope

**Component**	**F**	**Df1**	**Df2**	**P**
Motivation	0/152	1	25	0/700
Crossing	1/088	1	25	0/307

**Table3:** The Results of Multivariate Covariance Analysis to Examine the Effectiveness of the Intervention on Dimensions of Motivation and Crossing

**Component**	**Sum of square**		**DF**	**Mean of square**	**F**	**P**	${ƞ}^{2}$
Motivation	9/072	1		9/072	20/209	0/0001	0/468
Crossing	11/211	1		11/211	29/322	0/0001	0/560

## Discussion

The present study was conducted to examine the effectiveness of group positive parenting training on increasing hope and level of life satisfaction in mothers of children with autism. The results of this study showed that positive parenting training increased the total score of hope and its dimensions, motivation and crossing in mothers of the experimental group compared to the control group. This finding was consistent with that of previous studies (17, 19, 32). Studies show that parents of autistic children, especially mothers, are exposed to the problems related to mental health. Hopelessness and frustration are of the issues that mothers of autistic children face. There is significant evidence that high level of this frustration is associated with caring for the child with various disabilities (33) including autism. Based on Snyder's conceptualization (34), hope is a cognitive-behavioral structure that is consisted of the interaction of 3 main components including goal, the agent, and the crossing. Success in achieving the goals creates positive emotions and failure creates negative emotions. Hopeless individuals have less agents and crossings and when face an obstacle, they lose their motivation easily; thus, negative emotions develop in them causing depression, which has a negative effect on function of mothers with autistic children (35). The most common therapeutic method for these children is to help their parents learn the effective coping and problem- solving methods. Parents of these children need more training than the parents of healthy children, and thus training the parents is one of the methods that has a relative superiority to other methods (36). In this method, parents can have a significant role in implementing behavioral interventions in child's natural environment (37). In general, parents' training enables parents to help their children organize environmental condition, expand problem- solving skills, and cope with the disappointment. It also helps parents learn to show pleasant reaction to their children's endeavors and use regular and relaxing methods. Also, the results of the present study showed that group positive parenting training has increased life satisfaction in mothers of experimental group, and this result is consistent with that of previous studies (22, 27, 29). Life satisfaction is an arbitration process that with which the individuals evaluate their quality of life based on their unique criteria. Life satisfaction is not a permanent and objective characteristic and it is sensitive to temporary changes and is considered based on individuals' perceptions and attitudes. On the other hand, this disorder in children affects parents' life condition. Their parents often experience a lot of stress in their parenting role. Existence of disorders and behavioral problems in children contributes to the frustration of parents and makes changing roles and relationships of the parent - child difficult. Group positive parenting program is one of the 5 levels of family interventions that is especially used for the timely intervention for parents of 2-to12-year-old children that are suffering from or exposed to behavioral and emotional disorders. The aim of this program is to prevent the severe behavioral, emotional, and developmental problems through increasing awareness, skill, commitment, and confidence in parents (38). Costin et al. (39) found that training the parents and motivating them have a significant role in success and respond to treatment in children and parents. Researchers have found that training the parents reduces parenting stress and has a positive effect on the parent-child relationship. Hastings et al. (40) have examined the function of the families and coping strategies in families with autistic children. The results showed having such a child brings about a significant stress for the family and parents of these children have shown less life satisfaction, correlation, and family compatibility compared to the control group. Therefore, it is not strange that parents of children with autism have less psychological well-being and satisfaction. Mothers have lower mental health and experience more anxiety and stress compared to fathers (41). The results of various researches show that positive parenting training can give more satisfaction and happiness to mothers through presenting appropriate parenting strategies. Leong et al. (42) conducted a study on 91 parents of children with early conduct problems to examine the effectiveness of positive parenting training and found that after the intervention, participants reported significant reduction in child's behavioral problems and in inefficient parenting methods. Parents' competence also increased after the intervention. A study conducted by Telgen and Sanders showed that positive parenting program positively affects parents' life style, self-efficacy, satisfaction, compatibility, and relationships. This study also confirmed the effective role of positive parenting program for parents in helping them develop a positive loving relationship with their children through encouragement, attention, verbal compliments, and good communication. This program can also be used to deal with specific problematic behaviors in a constructive way and help parents to learn necessary skills to help their autistic children.

## Limitation

Lack of examining the long-term effectiveness of this training program was one of the limitations of this study. Longer follow ups are recommended in future. Second, study was limited to a group of mothers. Therefore, the study findings may not be generalizable to fathers. Future studies should consider the gender differences among both mothers and fathers on Group Positive Parenting Training program.

## Conclusion

Training is effective in managing and changing disruptive behaviors. On the other hand, increasing the awareness of mothers and their use of reward system to encourage their children and achieve the feeling of success, reduces behavioral problems in children, and thereby mothers’ stress reduces. The results of this study revealed that group positive training method had promising effects and increased hope and life satisfaction in mothers of children with autism. Thus, therapists and psychologists should pay more attention to the importance of group parenting training. 
